# Methotrexate-Induced Septicemia With Severe Pancytopenia and Diffuse Cutaneous Ulcerative Lesions

**DOI:** 10.7759/cureus.18069

**Published:** 2021-09-17

**Authors:** Iadarilang Tiewsoh, Biswajit Dey, Mary Chhangte, Monaliza Lyngdoh, Varsha Sathees

**Affiliations:** 1 General Medicine, North Eastern Indira Gandhi Regional Institute of Health and Medical Sciences, Shillong, IND; 2 Pathology, North Eastern Indira Gandhi Regional Institute of Health and Medical Sciences, Shillong, IND; 3 Dermatology, North Eastern Indira Gandhi Regional Institute of Health and Medical Sciences, Shillong, IND; 4 Internal Medicine, North Eastern Indira Gandhi Regional Institute of Health and Medical Sciences, Shillong, IND

**Keywords:** psoriasis, septicemia, ulcerative lesions, pancytopenia, toxicity, methotrexate

## Abstract

Methotrexate, a folate antimetabolite and one of the first few anti-neoplastic drugs, is now a commonly used drug in the treatment of many inflammatory disorders ranging from diseases like rheumatoid arthritis to psoriasis. The life-threatening toxicity of methotrexate in inflammatory diseases is not commonly encountered. Here we report a case of life-threatening multiorgan failure from methotrexate toxicity, which was given for skin lesions suspected to be psoriasis.

## Introduction

Methotrexate is a folate antimetabolite and the first few anti-neoplastic drugs that were administered for diseases like lymphoma and leukemia in high doses [1]. However, with the evolving evidence of its anti-inflammatory and immunomodulation properties, it has become one of the drugs of choice in many diseases like rheumatoid arthritis, small vessel vasculitis, and psoriasis [2-4]. The toxicity of methotrexate has been reported with both high and low doses and some risk factors are known to contribute to the toxicity [5-7].

Here we report a case of methotrexate toxicity with life-threatening multiorgan failure given to a patient with suspected cutaneous psoriasis.

## Case presentation

**Figure 1:**
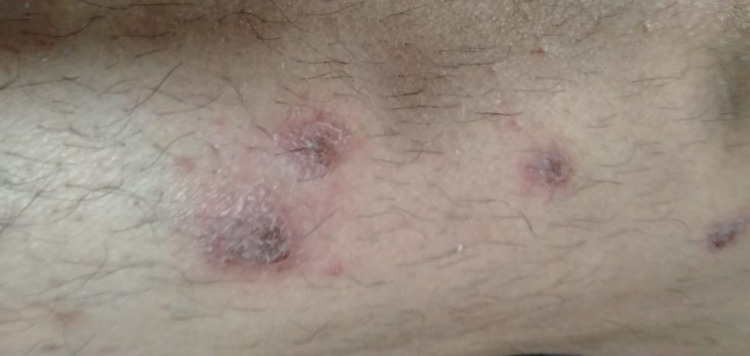
Initial lesion showing a blister on the upper part of the forearm and few vesicles with surrounding erythema and oedema on the lower part of the forearm.

**Figure 2:**
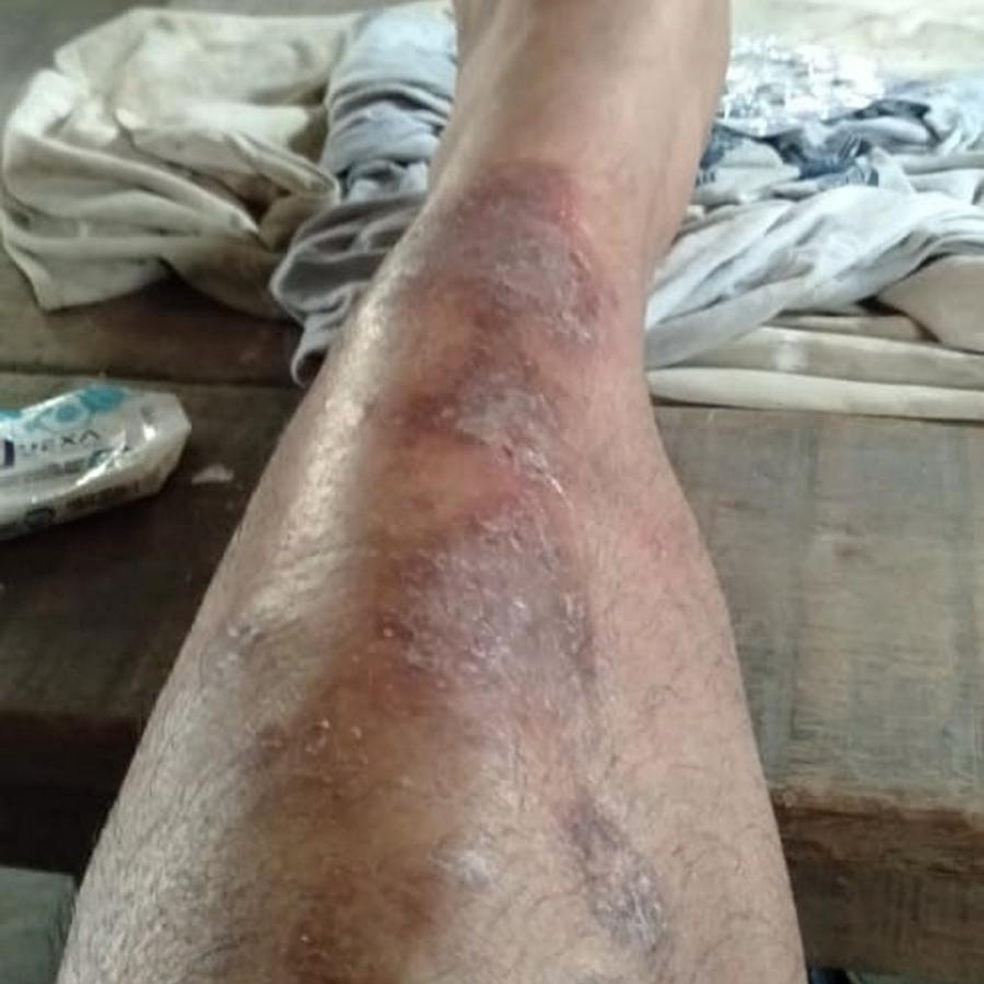
Few erythematous macules and scaly plaques with oedema on the lower limb.

**Figure 3:**
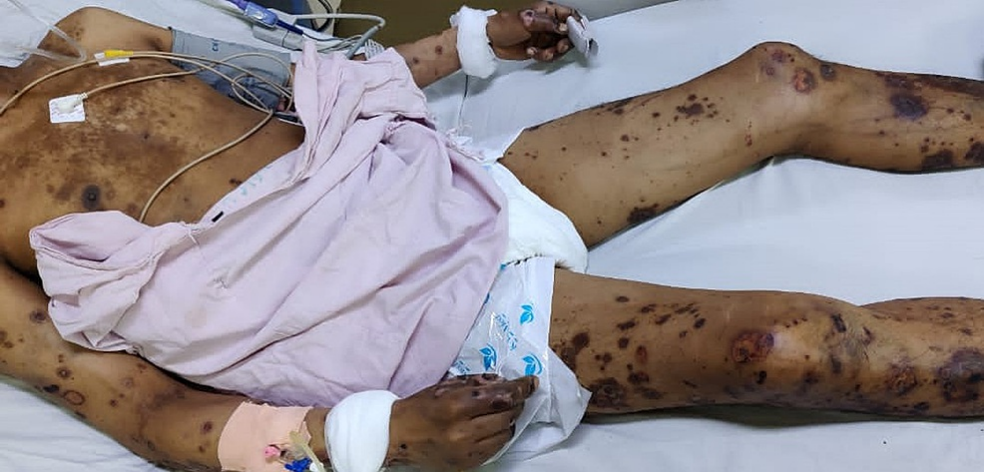
Multiple necrotic ulcers and hyperpigmented papules and macules distributed on the chest, the lower limbs and upper limbs bilaterally. Multiple erosions and necrotic ulcers on the back, some covered with crusts. Some of the lesions are healing with hyperpigmentation.

A middle-aged male of 46 years old, who is a chronic alcoholic, presented to the emergency department with multiple blisters and hemorrhagic crusting involving both upper and lower limbs and trunk for the last two months. The lesions started on the upper limbs as blisters and erosions following which the patient consulted a dermatologist (Figure 1). With a clinical suspicion of psoriasis, the patient was prescribed oral methotrexate weekly and topical steroids by a private practitioner. However after two weeks of the medication, the lesions increased resulting in multiple necrotic ulcers and hyperpigmented papules, macules distributed on the chest, the lower limbs and upper limbs bilaterally and erosions and necrotic ulcers on the back, some covered with crusts (Figure 2). This was followed by high-grade fever and difficulty in breathing. There was also a history of bleeding from the gums, mouth, and nasal cavity five days prior to admission. The patient went into a stage of altered sensorium and was rushed to our center for further management. On examination, the patient had mucositis of the buccal mucosa, active bleeding from the gums and nasal cavity. Examination of the skin lesions revealed multiple blisters and erythematous to hyperpigmented, scaly, and crusts on the neck, trunk, and limbs. There were multiple ulcers over these lesions with some of them were showing hemorrhagic crusts (Figure 3).

**Figure 4:**
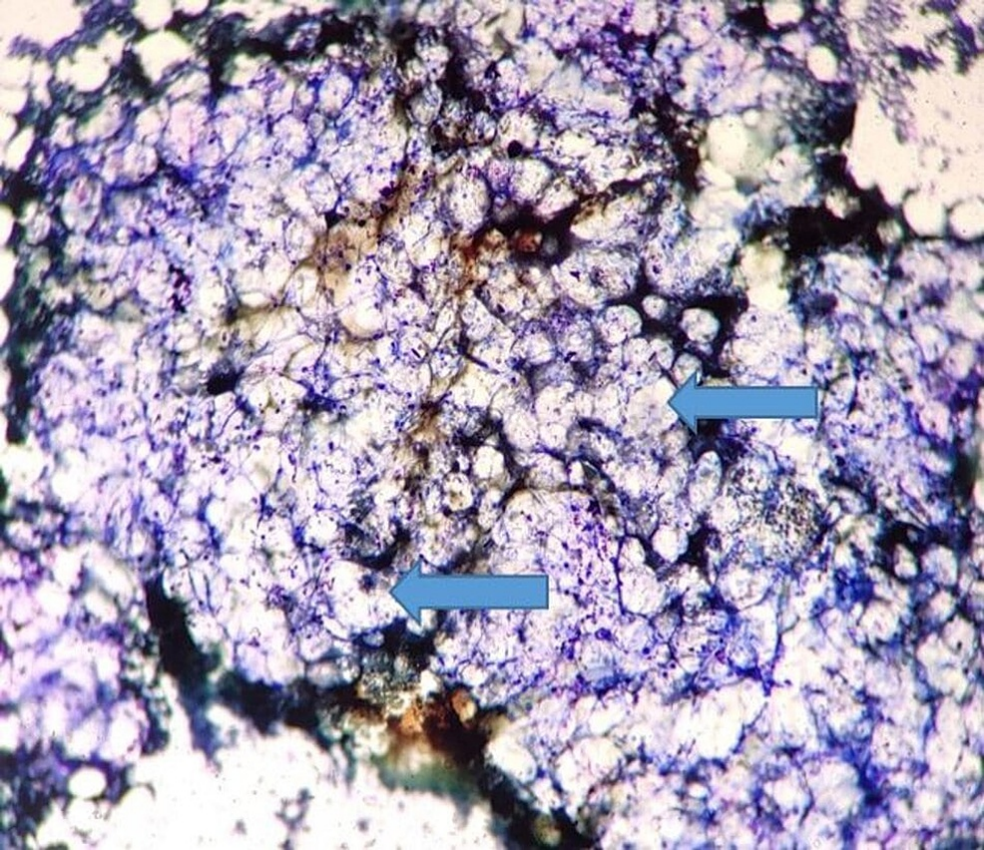
Bone marrow aspirate showing hypocellular particle with fat (arrows) replacing marrow particles (Leishman, 40x).

**Figure 5:**
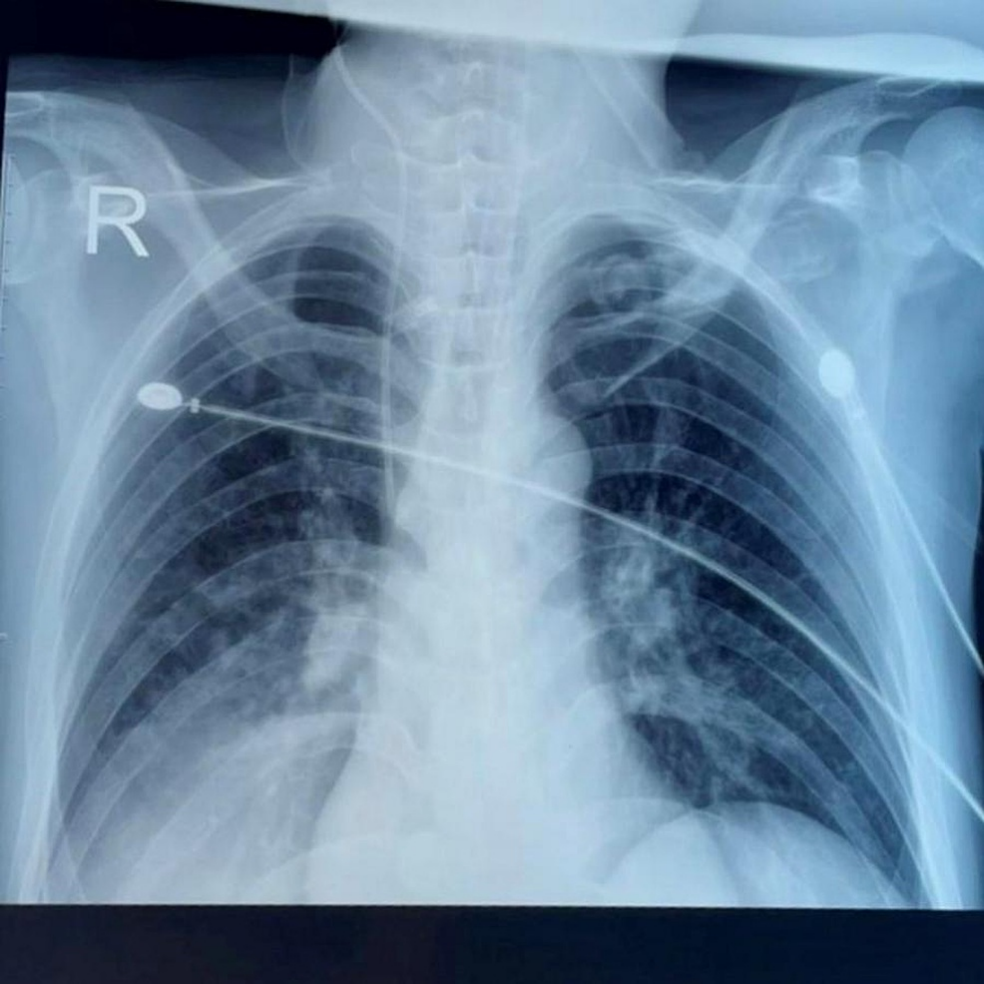
Chest X-ray showing right lower zone consolidation

**Figure 6:**
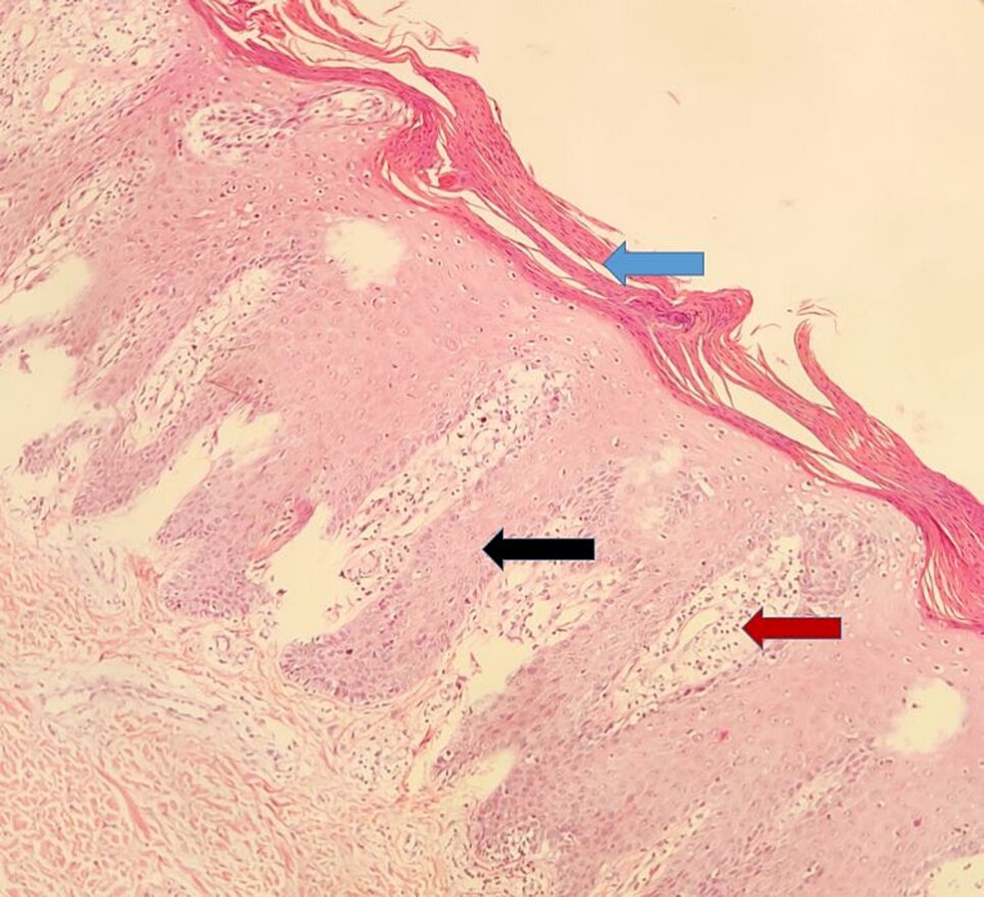
Skin biopsy showing parakeratosis of stratum corneum (blue arrow), psoriasiform hyperplasia (black arrow), and dilated capillaries in dermal papillae (red arrow) with suprapapillary thinning (H & E, 40x).

His baseline saturation was 60% at room air and 91% with high flow oxygen therapy. Systemic examination revealed basal crepitations on respiratory examination and Glasgow Coma score of 5/15 with no focal neurological deficits. A CT brain was down to rule out any intracranial hemorrhage which did not show any obvious abnormality. Laboratory investigations revealed severe pancytopenia with hemoglobin of 6 gm%, total leucocyte count (TLC) of 300/cumm, platelets of 5000/cumm, and a reticulocyte count of 0.08%. Peripheral blood smear showed macrocytic anemia with target cells, leukocytopenia with lymphocytosis, and severe thrombocytopenia. Biochemical parameter like serum lactate dehydrogenase (LDH) was raised (475 IU/L) and serum procalcitonin was elevated (25.24 ng/mL). His serum creatinine was 2.5 mg/dL and serum urea was 150 mg/dL. Bone marrow study revealed hypocellular marrow with marked suppression of erythroid, myeloid, and megakaryocytic cell lineages favoring myelosuppression. Residual erythroid and myeloid cell lineages also showed megaloblastic changes (Figure 4). His bone marrow culture and blood culture had significant growth of methicillin-resistant Staphylococcus aureus (MRSA) which was sensitive to vancomycin. His chest X-ray was suggestive of a right lower lobe consolidation (Figure 5). His skin biopsy taken from an intact lesion on the medial aspect of the left foot showed features of psoriasis (Figure 6). With the background history of worsening symptoms after the intake of methotrexate, the multiorgan dysfunction was suspected to be due to methotrexate toxicity, however, serum methotrexate estimation could not be carried out. All the investigations are tabulated in Table 1 and Table 2. Since low doses of methotrexate are unlikely to cause severe life-threatening complications a detailed history of the doses taken by the patient was obtained and it was found that the patient had consumed methotrexate daily at the dose of 10 mg for two weeks which was then followed by the increase of the cutaneous lesions and other complications. Based on the above clinical and laboratory findings with no other contributing factors, a diagnosis of methotrexate toxicity was made. Naranjo Algorithm-Adverse Drug Reaction Probability scale was 1 to 4.

**Table 1:** Haemotological and biochemical reports TLC: Total leucocyte count; DLC: Differential leukocyte count; ESR: Erythrocyte sediment rate; AST: Aspartate aminotransferase; ALT: Alanine transaminase; ALP: Alkaline phosphatase.

	1^st^ day of admission	5^th^ day of admission	10^th^ day of admission	On discharge
Hb (gm %)	6.2	4.7	9.1	9.2
TLC (per cumm)	300	2300	4900	5.7 x 10³
DLC (N/L/M/E)	38/50/06/06	70/24/05/01	73/18/08/01	70/23/04/03
Platelets (per cumm)	5000	23,000	30,000	250,000
Peripheral blood smear	Macrocytic anemia with target cells, leucocytopenia, lymphocytosis and severe thrombocytopenia			
ESR at the end of 1^st^ hour	66			
Reticulocytes	0.08			
Prothrombin time (sec)	16.3			
Activated partial thromboplastin time (aPTT) (sec)	40.8			
Fibrinogen levels (mg/dl)	267			
D-Dimer (ng/dL)	<250			
International normalized ratio (sec)	1.41			
Urea (mg/dL)	84	150	63	13
Creatinine (mg/dl)	2.5	2.3	1.3	0.9
Sodium (mmol/dL)	154.6	168.9	155.1	137
Potassium (mmol/dL)	3.79	3.69	4.59	3.66
Chloride (mmol/dL)	123.2	125.9	117.8	102
Total bilirubin (mg/dL)	2.5	1.0	1.5	0.7
Direct bilirubin (mg/dL)	1.4	0.4	0.7	0.2
AST (U/L)	59	35	43	19
ALT (U/L)	34	15	16	13
ALP (U/L)	98	102	162	141
Total protein (g/dL)	5.3	5.2	6.5	6.5
Albumin (g/dL)	2.4	2.2	2.1	3.4
LDH (IU/L)		475		
Procalcitonin (ng/mL)		25.24	5.04	1

**Table 2:** Biopsy and serology reports HCV: Hepatitis C virus; VDRL: Venereal disease research laboratory test; ANA: Antinuclear antibody; RT-PCR: Reverse transcription-polymerase chain reaction.

Bone marrow study	Hypocellular marrow with marked suppression of erythroid, myeloid, and megakaryocytic cell lineages favoring myelosuppression. Residual erythroid and myeloid cell lineages also showed megaloblastic changes.
Bone marrow culture	Staphylococcus aureus (methicillin-resistant)
Skin biopsy from the medial aspect of left foot	Psoriasiform hyperplasia with hypogranulosis and dilated capillaries with suprapapillary thinning confirming psoriasis
HBsAg / Anti-HCV	Negative
Anti-HCV	Non-reactive
VDRL	Negative
HIV	Non-reactive
ANA	Negative
RT-PCR for SARS-CoV-2	Negative

The patient was intubated and ventilated on the day of admission. He was managed on the line of severe sepsis with acute kidney injury, pneumonia, and pancytopenia with supportive skincare of the ulcerated lesions. The patient was started on intravenous leucovorin calcium, 25 mg every 6 hours on day 1, followed by 10 mg every 6 hours on days 2 and 3. Apart from intravenous antibiotic therapy and intravenous fluid, he received 2 units of packed red blood cells and 16 units of platelet concentrate due to upper gastrointestinal bleeding and bleeding from gums, nasal cavity, and buccal mucosa. Total parental therapy was continued for seven days. He responded to the medical treatment and on the 10th day of intensive care therapy, he was extubated and continued with supportive care. His lesions healed with the continuation of antibiotic therapy and daily dressing with povidone-iodine followed by the application of silver sulfadiazine cream. He was discharged on the 28th day of his hospital stay.

## Discussion

There have been case reports of methotrexate toxicities in the form of cutaneous skin lesions, acute renal failure, pancytopenia, pneumonitis, neurotoxicity [5-13]. Here we report methotrexate toxicity following over-dosage of methotrexate by the patient resulting in life-threatening multiorgan failure with severe pancytopenia, septicemia, acute kidney injury, and diffuse cutaneous skin lesions. Toxicities like myelosuppression, pancytopenia, acute renal failure, interstitial pneumonitis, hepatitis, gastrointestinal mucositis, leukoencephalopathy are severe forms usually associated with high doses and risk factors. The severity of methotrexate toxicity can vary from mild to severe forms.

Methotrexate is a folate antagonist and inhibitor of cellular proliferation. Cells with the highest turnover such as oral mucosa, gastrointestinal tract, and bone marrow cells are the most susceptible to its effect. Thus, mucositis develops when a patient's oral epithelial cells are affected. Myelosuppression in patients with methotrexate toxicity is explained by the same mechanism [14]. As a result, pancytopenia develops, which leads to increased bleeding, easy bruising, macrocytic red blood cells, and an increased risk of infections [15].

A varied range of skin disorders has been reported with methotrexate in high to low doses. These include Stevens-Johnson syndrome (SJS), toxic epidermal necrolysis (TEN), erythema multiforme, erythroderma, papular rash, photodermatitis reaction, epidermal necrosis, and cutaneous ulceration [7-9,16].

Ulceration of the psoriatic plaques in the skin due to methotrexate toxicity is uncommon. Two patterns of skin ulceration have been described in psoriasis patients, who are treated with methotrexate. In type I ulcers, the psoriatic plaques begin to erode shortly after commencing methotrexate treatment. Type II ulcers affect uninvolved skin observed with higher dose. The pathogenic mechanism was believed to be the direct toxicity of the drug in both types. Psoriatic plaques are initially painful and red, and then they develop superficial erosions [17].

When methotrexate is given to patients in high or low doses all preventive measures are to be taken before administering the drug. High doses of methotrexate are administered with maintaining a good urine output, urinary alkalinization, monitoring serum creatinine, electrolytes, pharmacokinetically guided leucovorin rescue therapy and serum methotrexate estimation. Toxicities from low doses of methotrexate are stomatitis, nausea, hepatitis, cutaneous eruptions, fever, macrocytosis, and myelosuppression which can be prevented by educating and monitoring the patient from time to time along with co-administration of weekly folic acid. Immediate withdrawal of methotrexate when toxicity is encountered is an important intervention with supportive therapy. Education about the proper dosage and the related toxicity should be explained to patients before administrating the drug.

In the present case, the patient had an overdose of the drug following which he suffered a severe form of bone marrow suppression, acute renal failure, and diffuse cutaneous ulcerative lesions with septicemia. Pancytopenia could have also been contributed by folate deficiency which might have persisted before the event with the patient being a chronic alcoholic. The skin lesions of the patient could be attributed to the direct toxicity of the methotrexate therapy.

## Conclusions

Methotrexate is a commonly used drug for many systemic inflammatory diseases and cutaneous lesions in clinical practice. The toxicity that our patient suffered was due to overdosage and resulted in life-threatening complications which if not timely managed the mortality is known to be very high. Hence the toxicities that can result from methotrexate should always be considered before initiating the drug therapy. It is important for all clinicians to carry out a detailed laboratory evaluation prior to initiation of the therapy, adequate education and close monitoring during the course of therapy to avoid the adverse drug events from methotrexate.
